# Activity of Potassium Channels in CD8^+^ T Lymphocytes: Diagnostic and Prognostic Biomarker in Ovarian Cancer?

**DOI:** 10.3390/ijms25041949

**Published:** 2024-02-06

**Authors:** Vivien Jusztus, Ghofrane Medyouni, Adrienn Bagosi, Rudolf Lampé, György Panyi, Orsolya Matolay, Eszter Maka, Zoárd Tibor Krasznai, Orsolya Vörös, Péter Hajdu

**Affiliations:** 1 Department of Biophysics and Cell Biology, Faculty of Medicine, University of Debrecen, Egyetem tér 1., H-4032 Debrecen, Hungary; jusztus.vivien@med.unideb.hu (V.J.); medyouni.ghofrane@med.unideb.hu (G.M.); bagosiadri@gmail.com (A.B.); panyi@med.unideb.hu (G.P.); orsolyavoros89@gmail.com (O.V.); 2 Department of Gynecology and Obstetrics, Faculty of Medicine, University of Debrecen, Egyetem tér 1., H-4032 Debrecen, Hungary; lampe.rudolf@med.unideb.hu (R.L.); orsolya.matolay@med.unideb.hu (O.M.); maka.eszter@med.unideb.hu (E.M.); krasznai.zoard@med.unideb.hu (Z.T.K.); 3 Division of Dental Biochemistry, Department of Basic Medical Sciences, Faculty of Dentistry, University of Debrecen, Egyetem tér 1., H-4032 Debrecen, Hungary

**Keywords:** ovarian cancer, tumor immunology, ion channel, CD8 lymphocytes, CRAC channel, potassium channel

## Abstract

CD8^+^ T cells play a role in the suppression of tumor growth and immunotherapy. Ion channels control the Ca^2+^-dependent function of CD8^+^ lymphocytes such as cytokine/granzyme production and tumor killing. Kv1.3 and KCa3.1 K^+^ channels stabilize the negative membrane potential of T cells to maintain Ca^2+^ influx through CRAC channels. We assessed the expression of Kv1.3, KCa3.1 and CRAC in CD8^+^ cells from ovarian cancer (OC) patients (*n* = 7). We found that the expression level of Kv1.3 was higher in patients with malignant tumors than in control or benign tumor groups while the KCa3.1 activity was lower in the malignant tumor group as compared to the others. We demonstrated that the Ca^2+^ response in malignant tumor patients is higher compared to control groups. We propose that altered Kv1.3 and KCa3.1 expression in CD8^+^ cells in OC could be a reporter and may serve as a biomarker in diagnostics and that increased Ca^2+^ response through CRAC may contribute to the impaired CD8^+^ function.

## 1. Introduction

Ovarian cancer (OC) is a common gynecological malignancy, which affects many women in the world. The incidence of OC is high, and it has a limited, <50%, 5-year survival rate [1] (incidence: 3.4% out of all cases for women and 22.5% for all gynecological cancers; source: https://gco.iarc.fr/ (accessed on 24 January 2024)). The detection of OC is problematic at the early stage; patients with OC are diagnosed in an advanced period of disease (stage III–IV), and by then, the cancer has generated metastasis in the upper abdomen. The critical point of survival is the stage of disease and the amount of residual lumps remaining after the surgical intervention. The most commonly used biomarker of non-mucinous epithelial ovarian cancer is the carcinoma antigene-125 (CA-125) as a serum marker, but it is not informative in mucinous cancers and germ cell tumors [2]. For platinum-based, drugs are available as the primary treatment, but in advanced-stage patients, this did not prove to be effective [3].

The immune system plays an important role in the fight against cancer. Specific antigen (even those presented on tumor cells) recognition by T cells is mediated by the TCR/CD3 (T cell receptor) complex, which in turn activates T lymphocytes [1,2]. CD8^+^ cytotoxic T cells are activated via antigen presentation by MHC I (major histocompatibility complex) receptors, and then they kill malignant cells: they induce target cell lysis, initiation of apoptosis by perforin and proteases (e.g., granzyme) and release IFN-γ (interferon gamma) and TNF (tumor necrosis factor) to stimulate macrophages [3]. This process involves ion channels, which can contribute to the Ca^2+^-dependent signaling pathway during activation. The ion channels of T lymphocytes, such as Kv1.3 (voltage-gated K^+^ channel 1.3), KCa3.1 (calcium-activated K^+^ channel 3.1) and CRAC (Ca^2+^ release-activated Ca^2+^ channel) channels, contribute to the Ca^2+^ signaling triggered by antigen presentation, which is necessary for T cell proliferation, however, other effector functions are also regulated by Ca^2+^ level, such as cytokine release, migration and even target cell-killing machinery [4]. Upon activation, the production of IP_3_ (inositol trsiphosphate) by PLCγ (phospholipase C) triggers a two-phase elevation of cytoplasmic Ca^2+^: the initial Ca^2+^ release from the ER (endoplasmatic reticulum) is followed by a more robust Ca^2+^ influx through the ORAI1 activated by ER resident STIM1 (stromal interaction molecule 1). Store-operated Ca^2+^ entry (SOCE) is maintained by the concerted operation of Kv1.3 and KCa3.1, as they sustain a constant driving force for the Ca^2+^ influx. The sustained Ca^2+^ allows NFAT (nuclear factor of activated T cells) to translocate to the nucleus and facilitates the transcription, e.g., interleukin-2, granzyme B (GrB), perforin [5,6,7].

The Kv1.3 ion channel was shown to be a key element of the pathway that regulates GrB production and secretion by CD8^+^ T cells [8]. An optimal intracellular Ca^2+^ level in CD8^+^ cells is required to maximize target cell-killing capacity, migration and lytic vesicles release, which is maintained by sufficient functional expression of Orai1 [9]. In addition, it was reported in mice models that upregulation of Kv1.3 expression in tumor-specific CD8^+^ cells impaired the melanoma tumor growth [10]. For NK cells (natural killer cells), a recent study also reported that interplay between CRAC and KCa3.1 channels is necessary for optimized target cell elimination [11,12]. KCa3.1 dysfunction in CD8 cells from cancerous patients limited their chemokine-induced migration when cells were exposed to high adenosine level [13].

Immune surveillance by the immune system often fails because tumor cells can reprogram vital pathways that ensure their survival [14]. Tumor-infiltrating lymphocytes (TILs) and CD8^+^ T cells are unable to efficiently perform effector functions and thus eliminate cancer cells due to various immunosuppressive mechanisms present in the tumor microenvironment (TME) [15]. The TME hinders CD8^+^ T cell effector functions via metabolic (elevated extracellular K^+^, H^+^, ATP and adenosine levels, hypoxia and increased acidity) and cellular components, such as programmed cell death ligand 1 (PD-L1) overexpression. With the increased extracellular K^+^, hypoxia has an inhibitory effect on Kv1.3 channels by inhibiting the channel’s activation. PD-L1 expressed on the surface of tumor cells binds to the PD-1 receptor on T cells, thereby triggering KCa3.1 inhibition via the PI3K (phosphatidylinositol 3-kinase) pathway. Adenosine binding to the A_2A_ receptor on T cells impairs the operation of KCa3.1 channels [16]. Nevertheless, tumor-resident regulatory T cells and tolerogenic dendritic cells suppress the anti-tumor capacity of infiltrating T lymphocytes [14].

In the present study, we evaluated the Kv1.3 and KCa3.1 expression of CD8^+^ peripheral blood-derived T cells from OC patients and healthy donors. We also determined the CRAC-dependent SOCE in these cells. The use of PBMCs (peripheral blood mononuclear cells) has a rationale to include a novel detection method of tumor malignancy with a non-surgical approach. Our results support the evidence that solid tumor growth could reprogram T cells (probably in a paracrine manner), which cancels T cell ability to migrate into the tumor and eliminate cancerous nodules.

## 2. Results

### 2.1. Malignant Tumor CD8^+^ Cells Have Low KCa3.1 and High Kv1.3 Conductance

Kv1.3 and KCa3.1 ion channels are important in the regulation of the Ca^2+^ response of T lymphocytes upon T cell receptor engagement by a specific antigen [13,17,18,19,20]. Ion channels play an essential role in effector functions of T cells and are essential in processes such as elimination of target cells such as cancerous cells in tumors [8,21,22]. The engineered T cell immune therapy failed in the treatment of human ovarian cancer: the T cells were unable to migrate into the tumor, which could be attributed to the change in the ion channel expression of patients’ own T cells [23].

**Figure 1:**
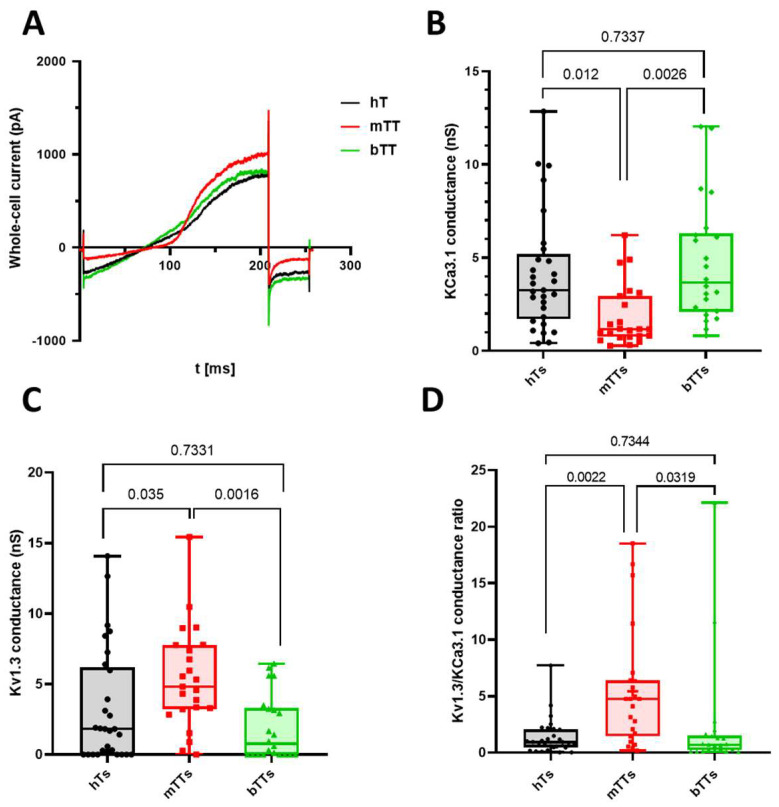
Different expression levels of KCa3.1 and Kv1.3 in CD8^+^ healthy donors and tumor patients. (**A**) Whole-cell current traces recorded in an hT, an mTT and a bTT upon a voltage-ramp protocol (see Section 4). (**B**,**C**) The box-plot of whole-cell conductance of KCa3.1 (**B**) and Kv1.3 (**C**) channels in healthy donors (hTs), malignant (mTTs) and benign (bTTs) tumor patients. (**D**) The box-plot for the ratio of Kv1.3 and KCa3.1 conductance in individual cells. In each graph, a symbol represents the average value determined for a cell. *p* values are indicated over the bars.

Hence, we wanted to evaluate the ion channels’ activity shaping the response of Ca^2+^. We assessed the functional expression level of Kv1.3 and KCa3.1 channels by electrophysiology and whole-cell conductance in activated T cells isolated from peripheral blood of healthy donors and patients with diagnosed tumor growth in ovarium (Figure 1A). Based on the pathological reports (that were exposed after the surgery), we categorized T cells from tumor patients into two groups: one with benign (bTT: benign tumor T cell) and the other with malignant (mTT: malignant tumor T cell) tumor. We observed that the Kv1.3 levels in mTTs were significantly higher as compared to cells isolated from healthy patients or benign tumor patients (whole-cell conductance values were 2.63 ± 0.88 nS for healthy T cells (hTs) (5 donors, *N* = 29), 5.33 ± 0.76 nS for mTTs (4 patients, *N* = 23), 2.46 ± 0.6 nS for bTTs (3 patients, *N* = 22), *p* values were 0.035 for hTs vs. mTTs and 0.0016 for mTTs vs. bTTs). For KCa3.1, the situation was upside-down: its conductance was far lower in mTTS, while in hTs and bTTs, the KCa3.1 level was the same (4.11 ± 0.58 nS for hTs, 1.82 ± 0.34 nS for mTTs, 4.7 ± 0.7 nS for bTTs, *p* values were 0.0123 for hTs vs. mTTs and 0.0026 for mTTs vs. bTTs). Furthermore, we found that the whole-cell capacitances of cells in different groups were not statistically different (whole-cell capacitance values were 6.5 ± 0.43 pF for hTs, 5.4 ± 0.41 pF for mTTs, 6.2 ± 0.52 pF for bTTs, *p* value was 0.175): these data show that activations of T cells were complete, and it is likely that the difference in ion channel expressions can modify functions other than activation of CD8 cells.

On the other hand, we determined the ratio of Kv1.3 and KCa3.1 conductance to map the cell-by-cell variance in these channels’ level (Figure 1D). As shown in Figure 1D, mTTs show a KCa3.1^low^Kv1.3^high^ phenotype, while hTs and bTTs were typically KCa3.1^high^ and Kv1.3^low^ (*p* values are 0.0022 between hTs and mTTs, 0.0319 between bTTs and mTTs).

### 2.2. mTTs Show Higher CRAC-Dependent Ca^2+^ Response

**Figure 2:**
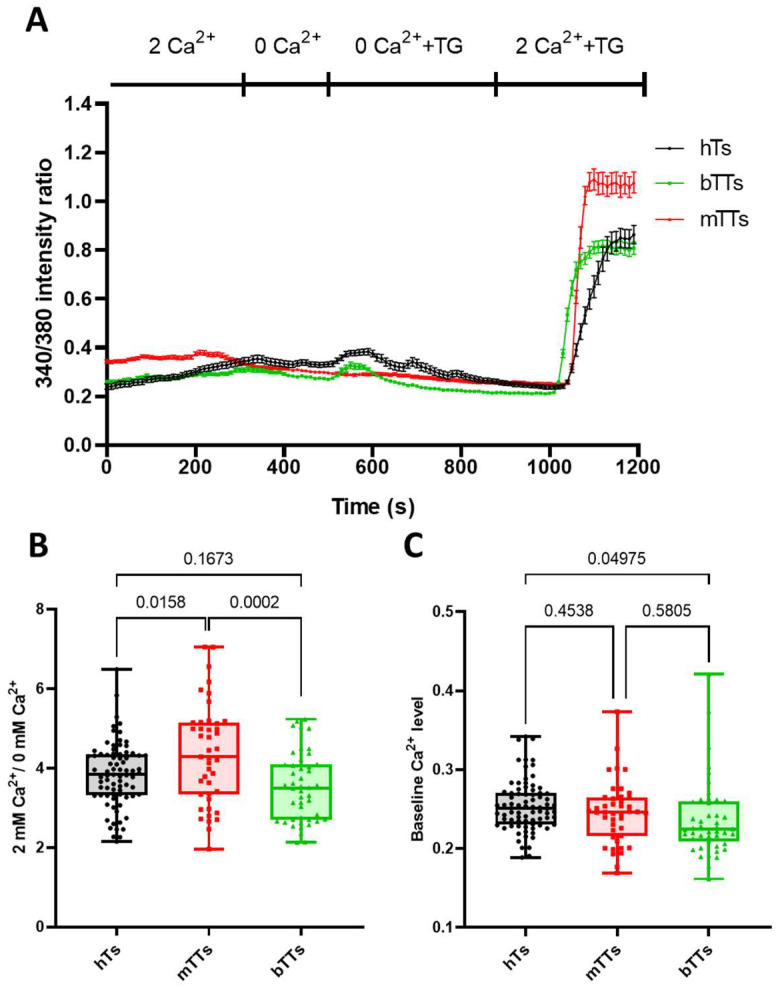
Thapsigargin-evoked Ca^2+^ response of mTTs is higher than for hTs and bTTs. (**A**) TG-induced Ca^2+^ response of hTs, mTTs and bTTs. Data points represent the average of 340 nm/380 nm intensity ratio of FURA-2 for several cells. (**B**) Box-plot of 2 Ca^2+^/0 Ca^2+^ intensity ratios for hTs, mTTs and bTTs. Each point represents a single-cell value. *p* values are indicated over the bars. (**C**) Box-plot of baseline Ca^2+^ intensity ratios for hTs, mTTs and bTTs. Each point represents a single-cell value.

As we described in the introduction, these three channels—Kv1.3, KCa3.1 and CRAC—can control the Ca^2+^-level changes during the early steps of antigen-dependent activation or in other effector functions. Hence, we assessed the store-operated Ca^2+^ entry of CD8 cells from healthy donors and tumor patients. Using the FURA-2 Ca^2+^ imaging method, the Ca^2+^-store ER was emptied with thapsigargin (TG, SERCA pump antagonist in the ER) in Ca^2+^-free solution, then Ca^2+^ was added back extracellularly to cells along with TG (Figure 2A).

As TG directly stimulates Orai1-STIM1 assembly to form CRAC, it cannot influence signaling steps via CD3/TCR; it only reports on the Ca^2+^ influx through SOCE and reflects the expression level of CRAC channels. Representative recordings of Ca^2+^ response of hTs, mTTs and bTTs are shown in Figure 2A: each curve represents the average of several cells measured in an experiment for a donor. Interestingly, the baseline in each group was the same, but the Ca^2+^ signal after adding back the Ca^2+^ into the extracellular solution was the highest for CD8^+^ cells isolated from malignant tumor patients. To quantify the Ca^2+^ response of cells, we determined the Ca^2+^ fold change: the ratio of the Ca^2+^ level before (baseline) and after (peak) Ca^2+^ re-addition was determined. As displayed in Figure 2B, the CRAC-related Ca^2+^ response of CD8^+^ cells was much higher in the mTT group as compared to hTs and bTTs. However, the baseline level of intracellular Ca^2+^ was not different for all groups (Figure 2C). 

## 3. Discussion

CD8 can also be expressed in the membrane of NK and γδ T cells, which could have been also studied in the present study. However, we should mention that lymphocytes are approximately 70–90% of PBMCs, of which at most (but normally lower) 10% are CD8^+^ NK cells. To our knowledge, they do not activate upon CD3/CD28, and they can be cultured for days but only in special medium (not used in the study). Gamma-delta T cells are mainly CD4^−^ and CD8^−^ and less than 5% of PBMCs. Based on these, we suppose that our panning protocol as well as labeling procedure ensure that we record electrophysiological data from CD8^+^ T cells. (One can easily estimate that recording of a CD8^+^ non-T cell has very low probability, which excludes their influence on the conclusion.)

Infiltration of CD8^+^ T along with NK (natural killer) cells into the tumor is inevitable for elimination of tumor cells. We have shown before that KCa3.1 channel activity is lower in CD8^+^ cells isolated from head and neck cancer (HNC) patients’ blood: it could be attributed to the reduced calmodulin expression or inhibition of adenosine through adenosine receptor A_2A_ [13,19,24,25]. Here, we demonstrated that KCa3.1 conductance is lower in CD8^+^ T cells that are from donors with malignant ovarian tumor but not those with benign ones. KCa3.1 channels regulate the chemokine-induced chemotaxis as well as random walk in T cells, both of which are hindered by the addition of adenosine via the A_2A_-PKA path. One may argue that these cells are suppressed by a relatively high concentration of adenosine produced by the tumor in the blood, however, this is not the scenario since adenosine’s half-life is very short, up to a few seconds. Use of our “model system”—namely PBMCs of patients—clearly demonstrates that impairment of CD8^+^ cell function does not happen only in the vicinity of the tumor: it can downregulate the KCa3.1 channels even at a distant location from the tumor to assure for itself survival. What is the benefit of KCa3.1 downregulation in CD8^+^ cells for the tumor? With low KCa3.1 activity, the migration capacity of CD8^+^ T cells is fairly reduced. This can explain why cytotoxic T cells are unable to infiltrate into the tumor: though they uncover the site of the tumor via a chemokine gradient, they are unable to migrate to the soma to eliminate the tumor’s core [13].

The immune responses of CD8^+^ cells partly rely on the Ca^2+^response, which is regulated by the Kv1.3 and KCa3.1 channels. Previously, it was shown by many groups that intra- and extracellular Ca^2+^ concentration definitely influence the target cell-killing efficiency of CD8^+^ cells: a moderate elevation in cytosolic Ca^2+^ level is required [9,11,12,26]. Here, we obtained that the CRAC-related response of mTTs is much higher compared to hTs or bTTs. We suppose that higher Ca^2+^ response in mTTs, and consequently the increased CRAC expression, counteracts their tumor cell-killing ability.

We must mention that our study has limitations. First, the number of donors/patients is limited in the present study, which is partly due to the benign and malignant classification of the patients’ group. Second, the descriptive characteristic of the analysis, which describes the difference in the whole-cell conductance of ion channels (Kv1.3 and KCa3.1) and CRAC-related Ca^2+^ response in the CD8^+^ cells. However, we suppose our results can contribute to the understanding of CD8^+^ T cell function and their ion channels’ role in cancerous diseases, and these data clearly show a noteworthy connection between the malignancy of the tumor and the ratio of Kv1.3 and KCa3.1 conductance. Furthermore, our data provide functional information (unlike immunofluorescent techniques) on ion channels at the single-cell level in T cells, which can be more useful in the design of novel immune therapies relying on T cell engineering.

## 4. Materials and Methods

### 4.1. Human Subject

The studies were performed on peripheral blood from unidentified ovarian cancer patients aged 44 to 71 years (4 malignant and 3 benign tumor patients). Inclusion criteria for the study were a positive diagnosis of OC confirmed by tissue biopsy and no chemotherapy or radiotherapy prior to the date of blood collection. The tumor stage was T2 and T3 for patients, with nodal status of N0 and N1. The data on the study subjects were collected and managed at the University of Debrecen, Faculty of Medicine, Department of Gynecology and Obstetrics. Peripheral blood was also drawn from 5 healthy female donors (HDs) in the age range of 40 to 55 years. Informed consent was obtained from all OC patients and HDs. The study and informed consent forms were approved by the Regional and Institutional Research Ethical Board of the University of Debrecen (RKEB/IKEB No.: 5091-2018).

### 4.2. PBMC Isolation and Activation

Peripheral blood mononuclear cells were isolated from whole blood as described previously [17]. Briefly, PBMCs were isolated by centrifugation of whole blood using a SepMate PBMC isolation tube (STEMCELL Technologies, Cambridge, MA, USA) with the Ficoll–Paque (GE Healthcare Bio-Sciences, Piscataway, NJ, USA) density gradient. PBMCs were separated from freshly drawn human blood, diluted twofold with HBSS ((Hank’s Balanced Salts Modified: (Sigma H4891, Ca^2+^- and Mg^2+^-free, 15 mM HEPES was added, pH = 7.4). We measured 15 mL of Histopaque-1077 into the SepMateTM PBMC isolation tube (STEMCELL Technologies, USA). The diluted blood was added to the SepMate tube up to the Ficoll layer. After centrifugation, the opalescent part, which contained peripheral mononuclear cells (PBMC), was transferred into a 50 mL tube and washed twice with HBSS and resuspended in RPMI medium (Sigma-Aldrich Ltd., Budapest, Hungary). The peripheral blood mononuclear cells (PBMCs) were maintained in RPMI medium (Sigma-Aldrich Ltd., Budapest, Hungary) supplemented with 10% Fetal Calf Serum, 15 mM HEPES, 2 mM L-glutamine, 1 mM Na-pyruvate and 200 units of streptomycin/penicillin. Activation was performed for 72 h in a 24-well cell culture plate coated with mouse anti-human CD3 and CD28 antibodies (both 10 µg/mL, BioLegend, San Diego, CA, USA).

### 4.3. Monoclonal Antibody Adhesion of CD8^+^ Cells

Activated PBMCs were labeled with mouse anti-human CD8 primary antibody (Biolegend, USA), and then the labeled cells were added to a bacterial-grade petri dish coated with goat anti-mouse IgG (Thermo Fisher Scientific Inc., Budapest, Hungary) [27]. Before electrophysiological recordings, cells were washed three times with extracellular solution.

### 4.4. Electrophysiology

KCa3.1 and Kv1.3 currents in CD8^+^ T cells were measured in a whole-cell voltage-clamp configuration at room temperature (23–25 °C) using a Multiclamp 700 B/Axopatch 200 A amplifier connected with an Axon Digidata1440 digitizer (Molecular Devices, Sunnyvale, CA, USA) to the personal computers. pClamp 10.6 software (Molecular Devices, Sunnyvale, CA, USA) was used for current recording and subsequent analysis. The extracellular bath solution contained the following (in mmol/L): 145 Na-aspartate, 5 KCl, 2.5 mM CaCl_2_, 1.0 MgCl_2_, 5.5 glucose, 10 HEPES (pH = 7.4). Patch-clamp pipette filling solution composition was as follows (in mM): 145 K-aspartate, 10 K_2_EGTA, 8.5 CaCl_2_, 2 MgCl_2_, 10 HEPES (free Ca^2+^ concentration 1 µM, pH = 7.2). Patch-clamp pipettes (4–6 MΩ) were pulled from GC150F-15 borosilicate capillaries (Harvard Apparatus, Kent, UK). Currents were elicited by a 200 ms ramp protocol ranging from −120 to +50 mV from a holding of −70 mV in every 15 s. The whole-cell conductance of KCa3.1 channels (G_KCa3.1_) and Kv1.3 (G_Kv1.3_) channels was calculated as we described before [16]. For each cell, the conductance was calculated as the average of three subsequent measurements.

### 4.5. Intracellular Ca^2+^ Measurements

Intracellular Ca^2+^ responses were measured as described previously [17]. Briefly, the activated PBMCs were labeled with Alexa-488 anti-mouse CD8 antibodies then loaded with 1 μM FURA-2-AM (Thermo Fisher Scientific Inc., Budapest, Hungary). The cells were perfused with 0 mM Ca^2+^ solution (143.3 mM NaCl, 4.7 mM KCl, 10 mM HEPES, 5.5 mM glucose, 1 mM MgCl_2_, 0.1 mM EGTA, pH 7.35) to check the cells’ integrity and then 1 μM thapsigargin (TG) (Thermo Fisher Scientific Inc.) containing 0 mM Ca^2+^ solution to deplete of ER and then 2 mM Ca^2+^ (143.3 mM NaCl, 4.7 mM KCl, 10 mM HEPES, 5.5 mM glucose, 2 mM CaCl_2_, 1 mM MgCl_2_, pH 7.35) containing 1 μM TG to activate intracellular Ca^2+^ elevation through CRAC.

### 4.6. Statistical Analysis

For statistical analysis and plotting, GraphPad Prism software was used (version 8.0.1). For multiple group comparison, one-way ANOVA or ANOVA on ranks was performed. Differences were considered significant at *p* < 0.05. Data were presented as mean ± standard error of the mean (SEM). Number of donors is denoted with “*n*”, and number of cells is designated with “*N*”.

## 5. Conclusions

In summary, we reported that the type of ovarian tumor (benign vs. malignant) defines the CD8^+^ T cells ion channel expression pattern. We suppose that lower KCa3.1 and higher CRAC activity contribute to the impaired CD8^+^ effector functions in tumor killing. Since total K^+^ conductance was the same in mTTs, the activation of these cells is not suppressed upon non-cancerous antigen stimulation. Finally, we suppose that reduced activity of KCa3.1 function in CD8^+^ cells of malignant tumor patients along with the elevated Kv1.3/CRAC activity could have a diagnostic implication.

## Data Availability

Data are contained within the article.
